# MicroRNA-146a-5p induces cell cycle arrest and enhances apoptosis in gastric cancer via targeting CDC14A

**DOI:** 10.3389/fcell.2023.1181628

**Published:** 2023-05-18

**Authors:** Piao Jiang, Bin Liang, Zhen Zhang, Bing Fan, Lin Zeng, Zhiyong Zhou, Zhifang Mao, Qing Lin, Weirong Yao, Qinglin Shen

**Affiliations:** ^1^ Department of Oncology, Jiangxi Provincial People’s Hospital, The First Affiliated Hospital of Nanchang Medical College, Nanchang, China; ^2^ The First Clinical Medical College, Nanchang University, Nanchang, China; ^3^ The First Affiliated Hospital of Nanchang Medical College, Institute of Clinical Medicine, Jiangxi Provincial People’s Hospital, Nanchang, China; ^4^ Department of Radiology, Jiangxi Provincial People’s Hospital, The First Affiliated Hospital of Nanchang Medical College, Nanchang, China; ^5^ Department of Thoracic Surgery, Jiangxi Provincial People’s Hospital, The First Affiliated Hospital of Nanchang Medical College, Nanchang, China

**Keywords:** microRNA-146a-5p, gastric cancer, CDC14A, cell cycle, apoptosis

## Abstract

**Objective**: The present study was designed to investigate the expression of miRNA-146a-5p in gastric cancer (GC) tissues and the paired nonmalignant counterparts, to explore the influences of miRNA-146a-5p on the cell biological behavior of MKN-28 cells (highly metastatic human gastric cancer cells), and to identify the function of abnormal expression of its target gene *cell division cycle 14 homolog A (CDC14A)* in GC.

**Methods:** We detected the expression of miRNA-146a-5p in formalin-fixed and paraffin-embedded (FFPE) GC tissues through microarray and quantitative real-time polymerase chain reaction (qRT-PCR). Then, we employed cell counting kit-8 (CCK-8) assays, cell cycle assays, and apoptosis analysis to uncover the latent function of miRNA-146a-5p in MKN-28 human GC cells. We also validated the target of miRNA-146a-5p via luciferase reporter assays.

**Results:** miRNA-146a-5p levels were examined in the majority of primary GC tissues and several GC cell lines. As a result, miRNA-146a-5p levels were significantly declined in the GC tissues and cells. In addition, miRNA-146a-5p demonstrated a straight act on its 3′-untranslated region (3′-UTR) of CDC14A mRNA, accordingly decreasing the contents of CDC14A mRNA as well as its protein expression. An inverse correlation between CDC14A and miRNA-146a-5p was observed.

**Conclusion:** The data suggest miRNA-146a-5p may contribute to inducing cell cycle arrest as well as prompting GC cell apoptosis via directly targeting CDC14A. Therefore, miRNA-146a-5p may be a potential indicator of the occurrence and development of GC.

## Introduction

Gastric cancer (GC) is the fifth most frequent malignancy in the world and the third leading cause of cancer-related death. Approximately 768,000 GC patients die annually worldwide (Smyth et al., 2020; Thrift and El-Serag, 2020; Ajani et al., 2022). Despite tremendous improvement in the study concerning oncogenesis and the development of GC, its intrinsic mechanisms are not well understood, thereby desperately compelling us to further investigate the prospective intrinsic mechanism unveiling GC.

MicroRNAs (miRNAs) are a class of short non-coding RNAs that regulate gene expression and manipulate stability as well as translation of mRNAs (Riahi Rad et al., 2021). miRNAs exert a vital part in numerous biological activities, including proliferation, differentiation, apoptosis, and motility (Saliminejad et al., 2019; Hussen et al., 2021; Liu et al., 2022). Dysregulated miRNAs have been observed in various tumors, such as, GC (Liu et al., 2021; Shi et al., 2021). miRNAs fulfill a role through binding to their miRNA recognition sequences which are common in the 3′-UTRs of target mRNAs, resulting in decreased translation and increased cleavage of target mRNAs, hence downregulating the protein expression downstream (Correia de Sousa et al., 2019; Pu et al., 2019). In addition, miRNAs are capable of serving as oncomiRs or oncosuppressor miRs (Ali Syeda et al., 2020; Miliotis and Slack, 2021; Smolarz et al., 2022). Typical instances in GC include miRNA-760, a kind of tumor repressor that targets G-protein-coupled receptor kinase interacting protein-1 (GIT1) (Ge et al., 2019), and miRNA-18, whose effects on gastric carcinogenesis are mediated by downregulating HMGB3 through targeting Meis2 (Zhang et al., 2022).

Cell division cycle 14 homolog A (CDC14A), a member of the dual specificity protein tyrosine phosphatase family, relates to the exit of cell mitosis and initiation of DNA replication, demonstrating its action on cell cycle control. Recent studies have reported that CDC14A might play an important role in cell adhesion (Chen et al., 2017) and apoptosis (Hu et al., 2019). However, the function of CDC14A in the cell cycle and apoptosis of GC remains unknown.

In our present study, we carried out miRNA microarray analysis and discovered that miRNA-146a-5p levels were significantly declined in GC tissues. Furthermore, we demonstrated that miRNA-146a-5p caused a reduction in CDC14A levels, leading to suppressed cell cycle and enhanced apoptosis. Altogether, the results suggest that miRNA-146a-5p may be conducive to cell cycle arrest and promotion of GC cell apoptosis, probably through targeting CDC14A.

## Materials and methods

### Tissue samples

The study was conducted in accordance with the principles of the Declaration of Helsinki. The approval for the study was granted by the Ethics Committee of Jiangxi Provincial People’s Hospital, The First Affiliated Hospital of Nanchang Medical College.

The formalin-fixed paraffin-embedded (FFPE) samples from 132 gastric cancer tissues and 66 matched noncancerous samples were obtained during surgery from January 2013 to May 2015 and then utilized after acquiring informed consent. The clinicopathological characteristics of the patients are summarized in Table 1. None of these patients accepted preoperative treatments like radiotherapy or chemotherapy. All patients underwent a definite histologic diagnosis of gastric cancer based on the clinicopathologic criteria according to the Eighth Edition of AJCC/UICC (2017). Invasive ductal carcinoma tissues, accounting for over 80% of the region of malignant epithelial cells, were identified using a microscope. Paired noncancerous samples were confined within 2 cm of tumor tissues and were not allowed to contain any tumor cells. Four pieces of 20-μm-thick segments were cut from each FFPE tissue block and harvested into a 2-mL RNA enzyme-free tube.

**TABLE 1:** Relationship of expression of miRNA-146a-5p in GC and clinicopathological characteristics.

Characteristic	Number (n = 132)	miRNA-146a-5p expression		*p-value*
		High (n = 40)	Low (n = 92)	
Age (years)				
<60	87	29	58	0.228
≥60	45	11	34	
Gender				
Male	81	24	57	0.721
Female	51	16	35	
Tumor size (cm)				
<5	89	29	60	0.367
≥5	43	11	32	
Tumor site				
Upper	30	3	27	0.001
Middle/lower	102	37	65	
Lauren’s classification				
Intestinal	72	8	64	<0.001
Mixed/diffuse	60	32	28	
Histologic differentiation				
Well/moderate	48	20	28	0.014
Poor and unknown	84	20	64	
Depth of invasion				
T1/T2	59	32	27	<0.001
T3/T4	73	8	65	
TNM stage				
I/II	52	22	30	0.003
III/IV	80	18	62	
Lymph node metastasis				
N0	42	19	23	0.002
N (+)	90	21	69	

### RNA preparation and qRT-PCR

We extracted total RNA from GC FFPE samples utilizing miRNeasy FFPE Kit (QIAGEN, Germany) following manufacturer’s instruction. Then, we used the NanoDrop 2000 spectrophotometer (Thermo Scientific, Germany) to determine the concentration and purity of all RNA extracts.

miRNA-146a-5p levels were determined utilizing stem–loop reverse transcription and qRT-PCR according to the previous literature (Jung et al., 2020). All reagents and consumables were purchased from Thermo Scientific (Germany). We carried out qRT-PCR three times and calculated by using the 2^−ΔCT^ method (Calin and Croce, 2006; Damanti et al., 2021), where ΔCt = Ct_miRNA-146a-5p_-Ct_U6_. CDC14A mRNA levels were tested three times, and the results were processed utilizing the 2^−ΔΔCT^ method, where ΔΔCt = ΔCt _miRNA-146a-5p_-ΔCt _NC_ and ΔCt = Ct _CDC14A_-Ct _β-actin_. All primers employed in stem–loop reverse transcription together with qRT-PCR are summarized in Table 2.

**TABLE 2:** Primers utilized for stem–loop RT as well as qRT-PCR.

Primer	Sequence
U6-F	5′-CTC​GCT​TCG​GCA​GCA​CA-3′
U6-R	5′-AAC​GCT​TCA​CGA​ATT​TGC​GT-3′
miRNA-146a-5p-RT	5′-GTC​GTA​TCC​AGT​GCA​GGG​TCC​GAG​GTA​TTC​GCA​CTG​GAT​ACG ACCACAAACC-3′
miRNA-146a-5p-F	5′-GCT​AGC​AGC​ACA​TAA​TGG​TTT​GTG-3′
miRNA-146a-5p-R	5′-GTG​CAG​GGT​CCG​AGG​TAT​TC-3′
CDC14A-F	5′-TAC​AAA​AGG​ACA​TCC​AAG​AGC​AG-3′
CDC14A-R	5′-TGG​CAC​CCG​AAG​ACA​AAG​A-3′

F, forward; R, reverse; RT, reverse transcription.

### miRNA microarray analysis

Microarrays were operated in four GC samples as well as four paired paracarcinomatous samples. Affymetrix miRNA 3.0 technology platforms, incorporating 1,733 mature human miRNAs, were adopted. Then, total RNA was spiked utilizing MicroRNA Spike-In Kit (Agilent Technologies), labeled using miRNA Complete Labeling and Hyb Kit (Agilent Technologies), hybridized to human miRNA microarrays, washed, and stained, followed by fluorescent signal intensities detection through the Affymetrix Scanner 3000. All of the aforementioned concrete steps were conducted in line with their manufacturer’s protocols.

Expression Console 1.3.1 (Affymetrix) was used for dissecting images to gain primitive information, followed by RMA normalization. Then, GeneSpring 12.5 (Agilent Technologies) was used as our analytical and visualization tool for microarrays. Furthermore, we adopted probes that possess flags in “P" in at least one group of the whole samples for a thorough study. Divergently expressed miRNAs were subsequently detected via fold change, where ≥ 2-fold differential expression implies upregulation of miRNA, along with a *p*-value ≤0.05 that implies downregulation of miRNA. In addition, we applied unsupervised hierarchical clustering to display the differences in the miRNA expression profiles amid the specimens.

### miRNA mimics and cell transfection

MKN-28, MGC803, SGC-7901, and GES-1 cell lines were purchased from ATCC (Rockville, MD) and cultured in complete media (Dulbecco’s modified Eagle media (DMEM) (Gibco BRL, Grand Island, New York)) supplemented with 15% fetal bovine serum (FBS), and then they were incubated at 37°C in 5% CO_2_. Negative control (NC) and miRNA-146a-5p mimics were generated and purchased from GenePharma (Shanghai, China). Utilizing Lipofectamine™ 2000 (Invitrogen), transfection of NC or miRNA-146a-5p mimics was performed separately on MKN-28 cells at a concentration of 100 nM, following the manufacturer’s guidance.

#### Cell cycle as well as apoptosis assays

Post-transfection 48 h, cells were washed twice with phosphate buffered saline (PBS) and then resuspended in ×1 binding buffer, followed by mixing with 500 μL propidium iodide (PI) [20 μg/mL PI, 50 μL/mL RNaseA, and 0.02% NP40 in PBS] with roughly 0.5 x 10^6^ cells at 4°C for 30 min. DNA quantification was implemented using the FACS Calibur instrument (Becton Dickinson, Mountain View, CA) and CellQuest software (Becton Dickinson). Through 12-h serum starvation and ∼18-h 2 mM hydroxyurea (HU) treatment, cells synchronized in G1/S transition. We identified cell apoptosis through fluorescein isothiocyanate (FITC) Annexin V Apoptosis Detection Kit (BD Pharmingen), following manufacturer’s suggested procedure. The whole experiment was conducted in triplicate.

#### Cell proliferation assay

Cells were loaded into a 96-well plate with a density of 3 × 10^5^ cells/mL by 0.1 mL/well, followed by miRNA-146a-5p mimics or NC transfection. We chose cell counting kit-8 (CCK-8) to test the absorbance values (OD_450nm_) using a microplate reader (BioTek) every 24 h for 5–7 consecutive days. All experiments were conducted in triplicate.

### Effects of miRNA-146a-5p on CDC14A levels

MiRNAorg, TargetScan, and PITA database were utilized for seeking the joint targets of miRNA-146a-5p in humans, with the pictures plotted using a Venn diagram as well as Cytoscape (version 3.7.0).

MKN-28 cells cultured normally *in vitro* were equally inoculated in six-well plates with 3 × 10^5^ cells in a volume of 1,000 μL media per well. The miRNA-146a-5p mimics and NC were transfected based on the suggested protocol utilizing Lipofectamine™ 2000. Then, we employed Western blotting (WB) to detect the CDC14A protein expression levels in the cells 48 h after transfection. Concrete steps of experiments demonstrating the effects of miRNA-146a-5p mimics and NC on CDC14A were performed as described in previous studies (Li et al., 2014; Kang et al., 2021).

#### CDC14A-3′-UTR luciferase reporter assay

We augmented the 3′-UTR elements of *CDC14A* gene from noncancerous tissues, accompanied by a straight sub-cloning stop codon of Renilla luciferase of psiCHECK2. Primers employed for amplification were as follows: CDC14A 3′-UTR-F: 5′-TAC​AAA​AGG​ACA​TCC​AAG​AGC​AG-3′ as well as CDC14A 3′-UTR-R: 5′-TGG​CAC​CCG​AAG​ACA​AAG​A-3’. **β**-actin was used as an internal control. **β**-actin-F: 5′GAT​CAT​TGC​TCC​TCC​TGA​GC-3′, **β**-actin-R: 5′-ACT​CCT​GCT​TGC​TGA​TCC​AC3’; the wild-type and mutant *CDC14A* 3′-UTRs were determined through sequencing. To perform luciferase screening, MKN-28 cells were inoculated into 96-well plates with approximately 5 x 10^3^ cells/well. After 24 h, MKN-28 cells were cotransfected by 25 pmol/well miRNA-146a-5p mimics or NC, together with 500 ng/well psiCHECK2-3′-UTR-CDC14A construct or psiCHECK2-3′-UTR-CDC14A mutation utilizing Lipofectamine™ 2000, according to their manufacturer’s guidelines. After 48-h cell incubation, firefly and Renilla luciferase activities were detected through dual-luciferase reporter assay (Promega). Then, we normalized Renilla luciferase activity to firefly luciferase activity. Similar to every experiment, we utilized the empty vector as the control, accordingly averaging out adjusted luciferase values. We discretionarily presume a value as “1,” considering it an antithesis to compare fold-differences of experimental values.

### Statistical analysis

Statistical difference was calculated utilizing Student’s t-test and analyzed using SPSS 26.0 (SPSS, Chicago, IL, United States). The chi-squared test was applied to assess the relationship between miRNA-146a-5p expression and clinicopathological parameters. Average outcomes were shown as mean ± standard deviation (SD). *p*-values < 0.05 (utilizing a two-tailed paired *t*-test) were regarded as suggestions of remarkable differences between the two groups of data.

## Results

### miRNA profiling in GC FFPE samples as well as qRT-PCR

**FIGURE 1:**
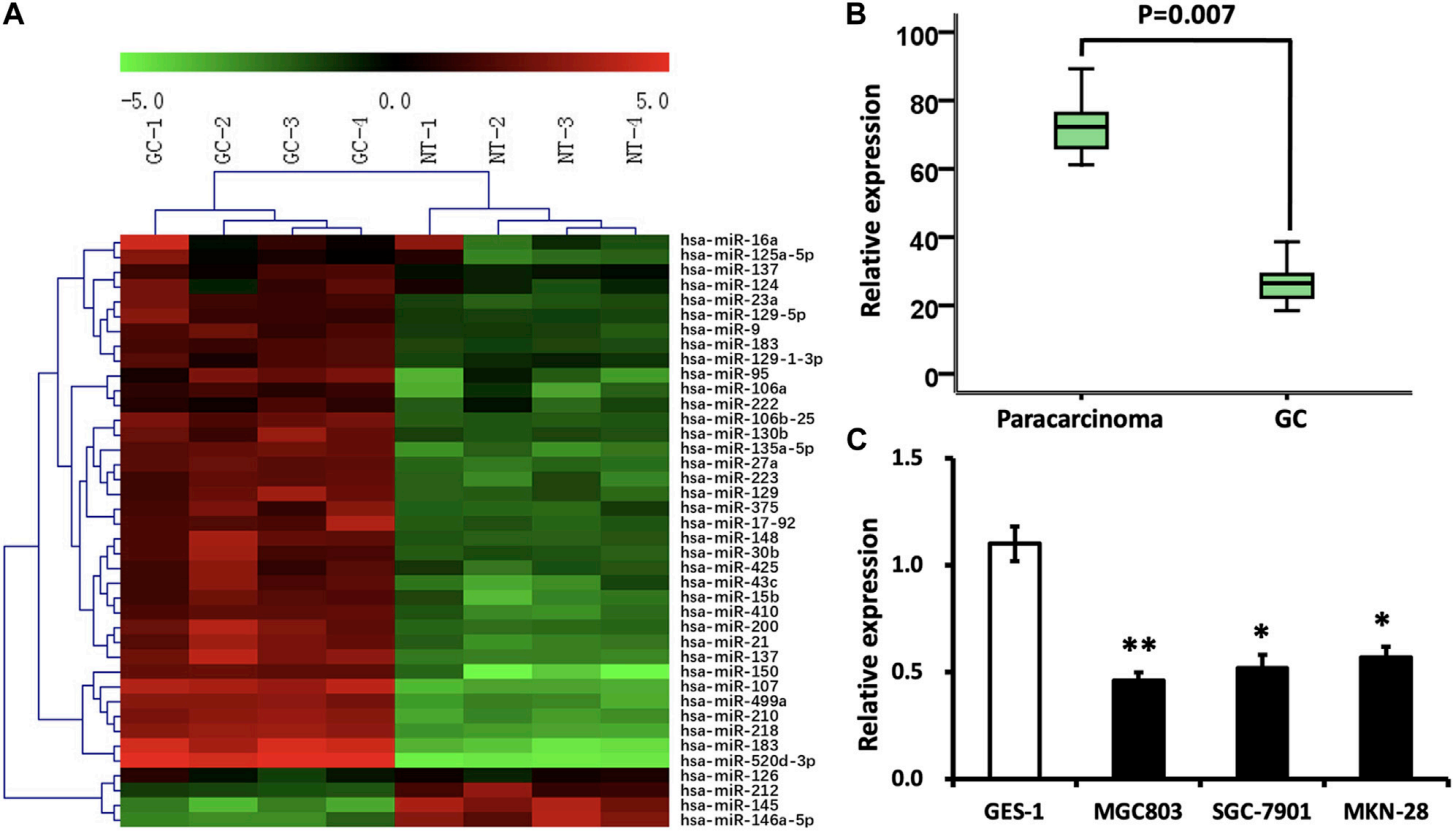
Differentially expressed miRNAs in GC. **(A)** Color scale demonstrates the comparative levels of miRNAs. “Red” implies high relative expression, and “green” implies low relative expression. **(B)** miRNA-146a-5p levels were reduced in GC samples (*n* = 132) in contrast to those in paracarcinomatous samples (*n* = 66) via stem–loop RT-PCR (*p* < 0.05). **(C)** Expression of miRNA-146a was analyzed in GC cell lines, MGC803, SGC-7901, and MKN-28, as well as normal gastric epithelial cells, GES-1. *p < 0.05, **p < 0.01 versus control.

To find out the miRNA which probably inhibits GC proliferation in terms of epigenetic regulation, miRNA microarray assay was conducted in four GC FFPE samples and four paracarcinomatous samples. Among the 40 variously expressed miRNAs, miRNA-146a-5p, miRNA-126, miRNA-212, and miRNA-145 were found to be decreased (Figure 1A). Nevertheless, the functions of miRNA-146a-5p in malignant tumors remain poorly understood. Therefore, our attention was predominantly concentrated on the miRNA-146a-5p.

To test and verify the alterations of the miRNA levels on the miRNA microarray, qRT-PCR was implemented to detect miRNA-146a-5p levels in 132 GC FFPE samples and 66 paracarcinomatous samples. In line with the outcomes demonstrated through miRNA microarray, the decline of miRNA-146a-5p was verified in GC FFPE samples (*p* < 0.05) (Figure 1B). In addition, the expression of miRNA-146a-5p was similarly remarkably decreased in GC cell lines, MGC803, SGC-7901, and MKN-28, in contrast to the noncancerous gastric epithelial cells, GES-1 (Figure 1C).

## Association between miRNA-146a-5p expression and clinicopathological characteristics

Comparing the miRNA-146a-5p expression with clinicopathological variables, we observed remarkable positive correlations between miRNA-146a-5p expression and tumor sites (*p* = 0.001), Lauren’s classification (*p* < 0.001), histologic differentiation (*p* = 0.014), invasion depth (*p* < 0.001), TNM stage (*p* = 0.003), and lymph node metastasis (*p* = 0.002) (Table 1).

### Cell apoptosis, cell cycle, and cell proliferation assays

**FIGURE 2:**
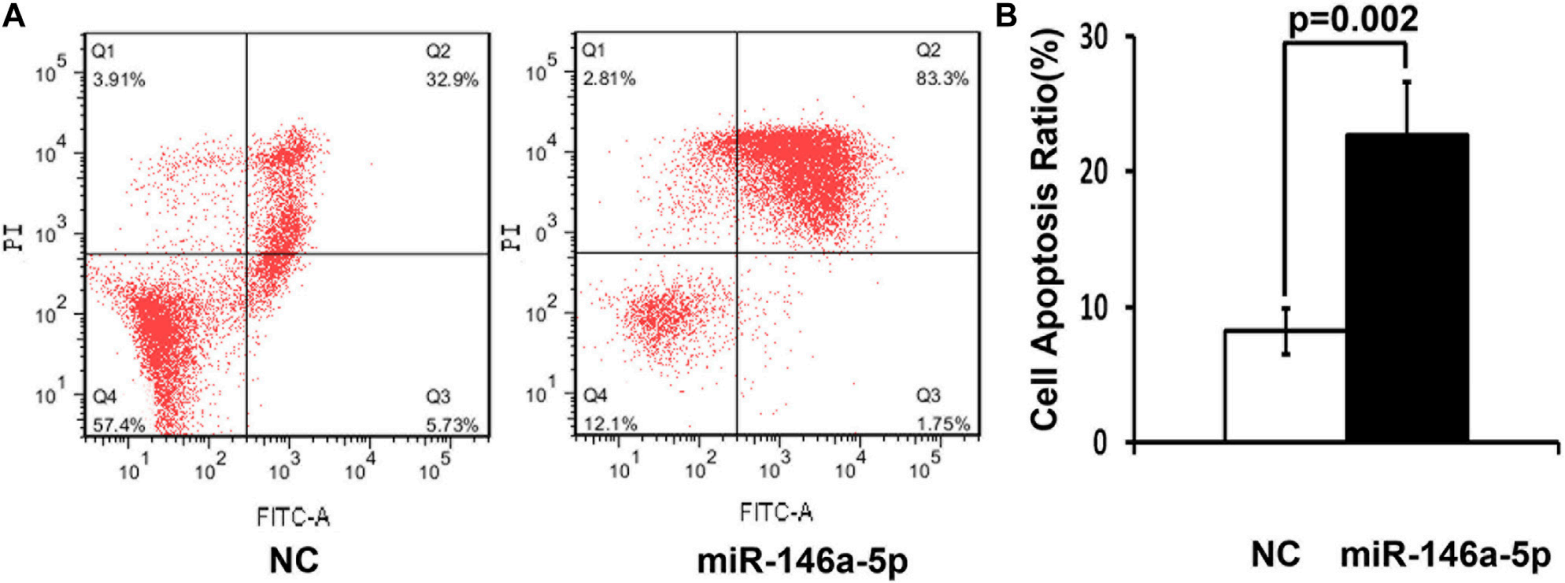
High miRNA-146a-5p levels enhance cell apoptosis. **(A)** 48 h after transfection by 100 nM miRNA-146a-5p mimics or NC, MKN-28 cells were stained utilizing Annexin V-FITC along with PI, followed by flow cytometry detecting apoptosis. **(B)** Semiquantitative analysis is shown. Transfection of MKN-28 cells by miRNA-146a-5p mimics led to remarkable alterations of increased apoptosis in contrast to NC (*n* = 3, mean ± SD) (*p* < 0.05).

MKN-28 cells were transfected by miRNA-146a-5p mimics or NC at the concentration of 100 nM. After 48 hours, MKN-28 cells were stained using Annexin V-FITC and PI, followed by flow cytometry detecting cell apoptosis. Our study exhibited that MKN-28 cells transfected by miRNA-146a-5p mimics led to dramatic alterations in the frequency of apoptosis, which is in contrast with the NC group (*p* < 0.05) (Figures 2A, B).

**FIGURE 3:**
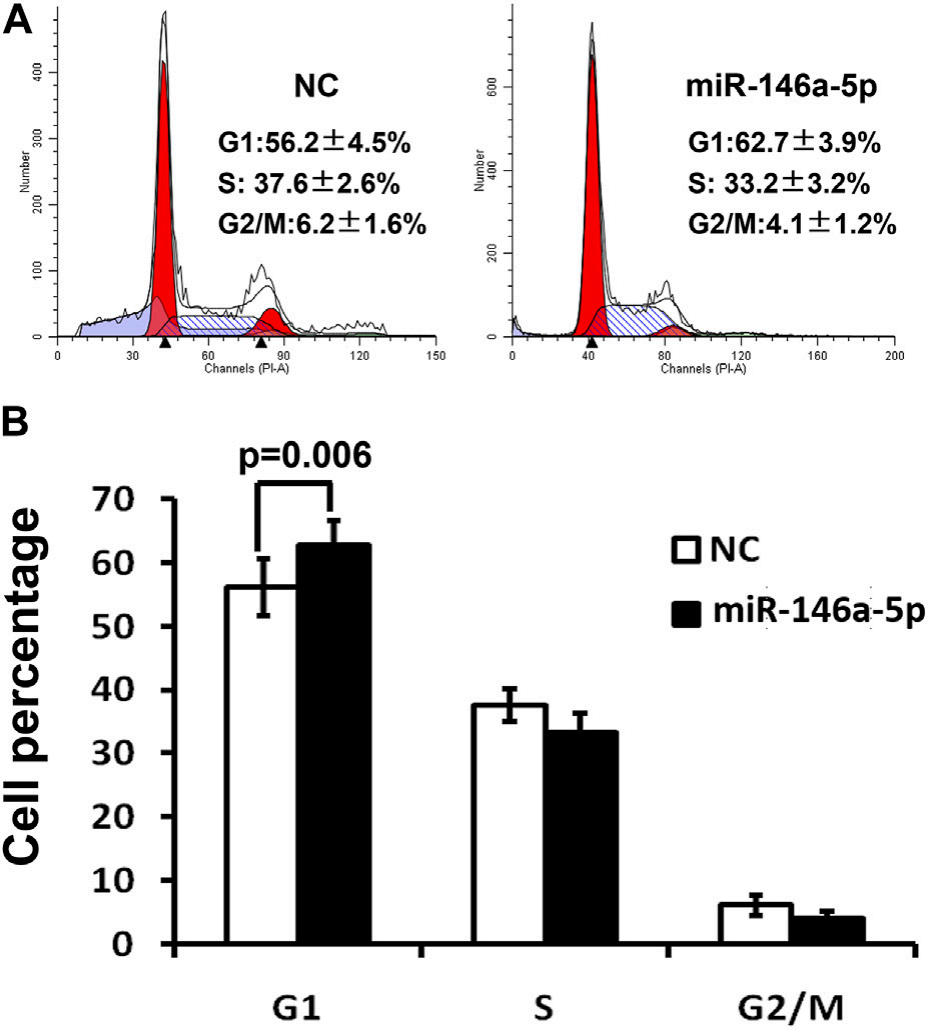
Cell cycle was detected via flow cytometry 48 h after transfection of MKN-28 cells by miRNA-146a-5p mimics or NC. **(A)** Flow cytometry demonstrates MKN-28 cell G1 phase arrest, as well as suppression of G1/S transition caused by increased miRNA-146a-5p in contrast to NC. **(B)** Semiquantitative analysis exhibited the relative proportion of cells at G1, S, and G2/M phases (*n* = 3, mean ± SD).

To validate that miRNA-146a-5p resulted in G1 arrest, MKN-28 cells transfected by miRNA-146a-5p mimics synchronized in the G1/S transition via serum deprivation as well as HU treatment. DNA quantification analysis was conducted upon releasing HU. These outcomes exhibited that all miRNA-146a-5p mimic-transfected cells started arresting at the G1 phase, suppressing their G1-to-S phase transition (*p* < 0.05) (Figures 3A, B).

**FIGURE 4:**
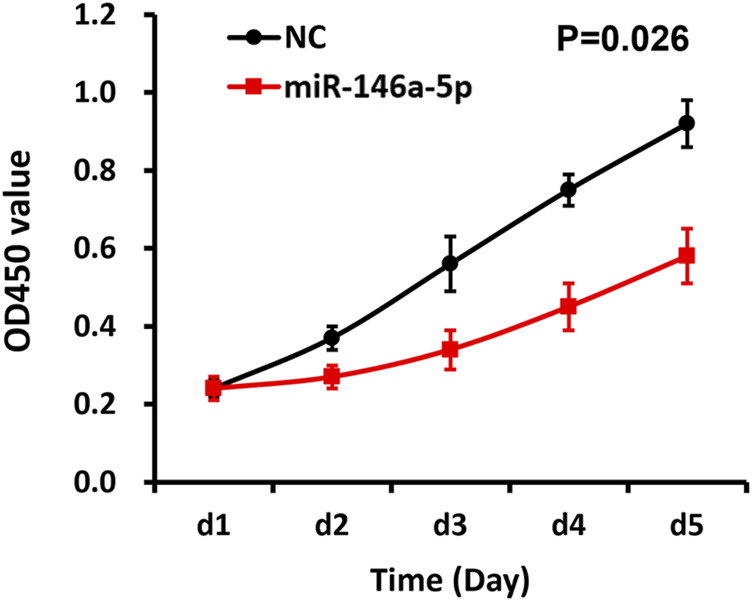
miRNA-146a-5p inhibits MKN-28 cell proliferation. Subsequent to transfection of MKN-28 cells by miRNA-146a-5p mimics or NC, absorbance values (OD_450nm_) were determined using CCK-8 assay every 24 h for 5 consecutive days (*n* = 3, mean ± SD) (*p* < 0.05).

Utilizing CCK-8 assay, our study demonstrated that the cell proliferation of the miRNA-146a-5p mimic-transfected group descended in contrast to the results of the NC group (*p* < 0.05), indicating that upregulated miRNA-146a-5p decreased MKN-28 cell proliferation (Figure 4).

### CDC14A acts as a target of miRNA-146a-5p

As is known, miRNAs generally exert a crucial influence on cellular behaviors by targeting pivotal downstream genes. To probe the latent intrinsic mechanisms elaborating why miRNA-146a-5p restrained GC cells, two calculation means were applied to assist in determining miRNA-146a-5p targets (Betel et al., 2008). Bioinformatics analysis suggested that 3′-UTR of the *CDC14A* gene incorporates a target site of miRNA-146a-5p, which was purely complementary to the 2–8 nt of miRNA-146a-5p.

**FIGURE 5:**
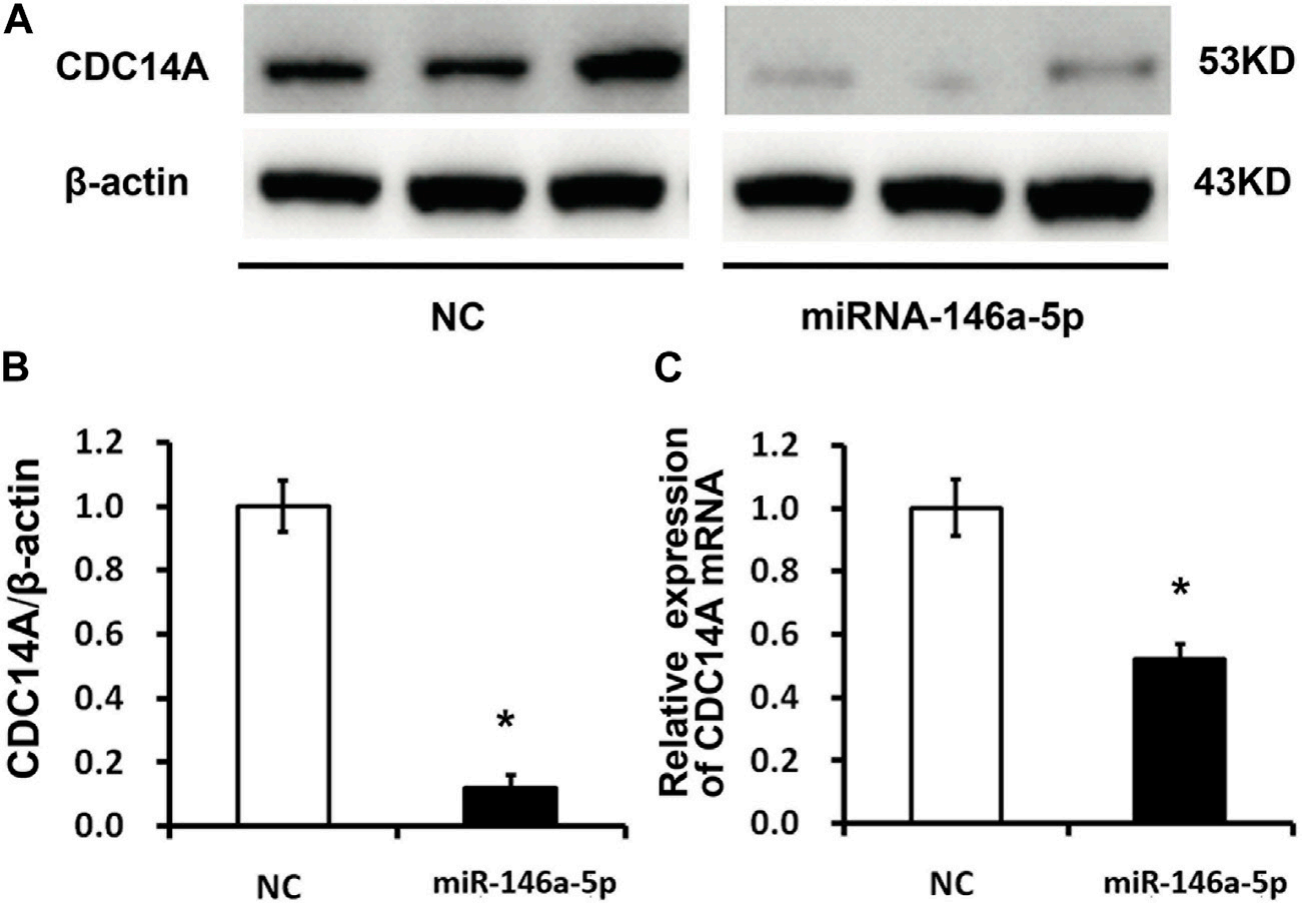
CDC14A targeted miRNA-146a-5p via binding to its 3′- UTR. **(A, B)** CDC14A protein levels were detected through WB experiments subsequent to transfection of miRNA-146a-5p mimics or NC into MKN-28 cells (*n* = 3, mean ± SD). **(C)** mRNA levels of CDC14A were detected via qRT-PCR subsequent to transfection of miRNA-146a-5p mimics or NC into MKN-28 cells. *p < 0.05, **p < 0.01 versus control.

In contrast to the NC group, mRNA levels of CDC14A identified by qRT-PCR declined in the miRNA-146a-5p mimic-transfected group (*p* < 0.05) (Figure 5C). Consistently, WB experiments on CDC14A protein levels exhibited remarkably reduced CDC14A levels in the miRNA-146a-5p mimic group compared with those in the NC group (*p* < 0.05) (Figures 5A, B).

**FIGURE 6:**
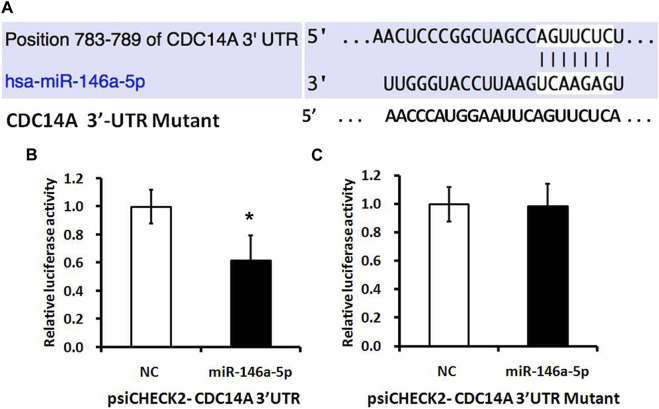
miRNA-146a-5p targets *CDC14A* gene. **(A)** Presumptive miRNA-146a-5p binding site in the *CDC14A* gene 3′-UTR. **(B, C)** Luciferase activity assays of luciferase reporters with wild-type or mutant *CDC14A* 3′-UTR were carried out subsequent to the Renilla luciferase activity (*n* = 3, mean ± SD). *p < 0.05 versus control.

A luciferase reporter vector with the speculative CDC14A-3′-UTR target spot for miRNA-146a-5p downstream of psiCHECK2-CDC14A 3′-UTR was fabricated. Likewise, we prepared a 10-bp mutant “psiCHECK2-CDC14A 3’-UTR” whose mutation sites were restricted in the seed region (psiCHECK2-CDC14A 3′-UTR mutating) (Figure 6A). Luciferase reporter vector, accompanied by miRNA-146a-5p mimics or NC, was co-transfected into MKN-28 cells. It turned out that a considerable decline of relative luciferase activity was observed in the experimental group, of which psiCHECK2-CDC14A 3′-UTR was co-transfected by miRNA-146a-5p mimics rather than NC (Figure 6B). Nevertheless, the inhibition was abrogated via mutating the 3′-UTR where miRNA-146a-5p binds to (psiCHECK2-CDC14A 3′-UTR mutating), hence disturbing the interaction between miRNA-146a-5p and *CDC14A*-3′-UTR (Figure 6C).

## Discussion

miRNAs have been proven by abundant studies to exert a significant effect on the progression of the malignant phenotype of GC, featuring increased cell survival, proliferation, tumor angiogenesis, dedifferentiation, and generation of cell stemness (Dragomir et al., 2022; Smolarz et al., 2022). Identification of the biological importance of disordered miRNAs in GC cells fills a few dissociated gaps formerly reported and offers a model system by which the effects of miRNA on tumorigenesis as well as tumor progression may be further apprehended. Furthermore, it has been validated that alterations in the expression level of specific miRNAs may stand for a novel type of an indicator of the existence and deterioration of GC, hence possessing diagnostic or therapeutic merits (Condrat et al., 2020; Wu et al., 2020; Ho et al., 2022).

As we all know, dysregulated miRNAs exert a crucial influence on carcinogenesis and cancer progression (Matsuyama and Suzuki, 2019). We demonstrated that miRNA-146a-5p was downregulated in GC tissues according to our results. Furthermore, we also observed that miRNA-146a-5p may function as an oncosuppressor miR to suppress cell proliferation through intercepting the G1/S transition of GC cells. That is to say, decreased miRNA-146a-5p levels in GC cells and tissues probably function to facilitate cell multiplication via attenuating the cell cycle.

Previous reports indicated that miRNA-146a-5p affects the apoptosis and proliferation of cells by regulating the signaling pathways (Zhang et al., 2015; Noorolyai et al., 2020; Noorolyai et al., 2021). Substantial studies suggested that upregulation of miRNA-146a-5p restricted cell growth, confined migration, arrested cells at the G1 phase, and promoted cellular apoptosis (Huang et al., 2019; Zhu et al., 2020; Ding et al., 2021). Therefore, our results are consistent with previous reports. CCK-8 assays and flow cytometry analysis were employed in our research to identify the roles of miRNA-146a-5p in MKN-28 cells. These outcomes of our research coincide with preceding studies concerning the actions of miRNA-146a-5p in other kinds of cancers.

CDC14A aberrant expression can lead to spindle structural damage, the separation of chromosomes, and cytoplasmic abnormal segregation (Mailand et al., 2002; Villarroya-Beltri et al., 2022). Previous research studies showed that CDC14A overexpression leads to chromosome structural damage and inhibits the regeneration of chromosomal microtubules, thus influencing the spindle shape and resulting in its dysfunction (Vázquez-Novelle et al., 2010; Hu et al., 2019). Therefore, CDC14A may play an important role in every step of mitosis (Ji et al., 2012; Ovejero et al., 2018; Huang et al., 2020). As a result, CDC14A can serve as one of the most significant molecules involved in regulating the cell cycle through the mechanism of regulating the expression of multiple related genes. We discovered that miRNA-146a-5p induced the decline of CDC14A expression, thus causing suppressed cell cycle progression and enhanced cell apoptosis. To sum up, we extrapolate that miRNA-146a-5p affects MKN-28 cell proliferation and apoptosis through manipulating the expression of CDC14A.

Collectively, miRNA-146a-5p levels were decreased in GC cells and tissues, and a converse influence on CDC14A protein levels was confirmed. miRNA-146a-5p may be involved in manipulating cell cycle arrest as well as prompting cell apoptosis of GC cells, probably through directly targeting CDC14A. As a result, miRNA-146a-5p may be implicated in the occurrence and development of GC and may be conducive as a novel prognostic marker and therapeutic tool.

## Data Availability

The original contributions presented in the study are included in the article/Supplementary Material; further inquiries can be directed to the corresponding authors.
